# Abdominal adipose tissue and liver fat imaging in very low birth weight adults born preterm: birth cohort with sibling-controls

**DOI:** 10.1038/s41598-022-13936-1

**Published:** 2022-06-14

**Authors:** Juho Kuula, Jesper Lundbom, Antti Hakkarainen, Petteri Hovi, Helena Hauta-alus, Nina Kaseva, Samuel Sandboge, Johan Björkqvist, Johan Eriksson, Kirsi H. Pietiläinen, Nina Lundbom, Eero Kajantie

**Affiliations:** 1 Department of Radiology, HUS Medical Imaging Center, University of Helsinki and Helsinki University Hospital, Helsinki, Finland; 2 Population Health Unit, Finnish Institute for Health and Welfare, Helsinki and Oulu, Finland; 3 Children’s Hospital, Pediatric Research Center, University of Helsinki and Helsinki University Hospital, Helsinki, Finland; 4 Research Program for Clinical and Molecular Metabolism (CAMM), Faculty of Medicine, University of Helsinki, Helsinki, Finland; 5 PEDEGO Research Unit, MRC Oulu, Oulu University Hospital and University of Oulu, Oulu, Finland; 6 Psychology/Welfare Sciences, Faculty of Social Sciences, University of Tampere, Tampere, Finland; 7 Department of General Practice and Primary Health Care, University of Helsinki, Helsinki, Finland; 8 Folkhälsan Research Center, Helsinki, Finland; 9 Singapore Institute for Clinical Sciences, Agency for Science, Technology and Research (A*STAR), Singapore, Singapore; 10 Human Potential Translational Research Programme and Department of Obstetrics and Gynecology, Yong Loo Lin School of Medicine, National University of Singapore, Singapore, Singapore; 11 Obesity Research Unit, Research Program for Clinical and Molecular Metabolism, Faculty of Medicine, University of Helsinki, Helsinki, Finland; 12 Obesity Center, Endocrinology, Abdominal Center, Helsinki University Hospital and University of Helsinki, Helsinki, Finland; 13 Department of Clinical and Molecular Medicine, Norwegian University of Science and Technology, Trondheim, Norway; 14 Children’s Hospital, Helsinki University Hospital and University of Helsinki, Helsinki, Finland

**Keywords:** Paediatric research, Outcomes research, Epidemiology, Obesity

## Abstract

Preterm birth at very low birth weight (VLBW, < 1500 g) is associated with an accumulation of cardiovascular and metabolic risk factors from childhood at least to middle age. Small-scale studies suggest that this could partly be explained by increased visceral or ectopic fat. We performed magnetic resonance imaging on 78 adults born preterm at VLBW in Finland between 1978 and 1990 and 72 term same-sex siblings as controls, with a mean age of 29 years. We collected T1-weighted images from the abdomen, and magnetic resonance spectra from the liver, subcutaneous abdominal adipose tissue, and tibia. The adipose tissue volumes of VLBW adults did not differ from their term siblings when adjusting for age, sex, and maternal and perinatal factors. The mean differences were as follows: subcutaneous − 0.48% (95% CI − 14.8%, 16.3%), visceral 7.96% (95% CI − 10.4%, 30.1%), and total abdominal fat quantity 1.05% (95% CI − 13.7%, 18.4%). Hepatic triglyceride content was also similar. VLBW individuals displayed less unsaturation in subcutaneous adipose tissue (− 4.74%, 95% CI − 9.2%, − 0.1%) but not in tibial bone marrow (1.68%, 95% CI − 1.86%, 5.35%). VLBW adults displayed similar adipose tissue volumes and hepatic triglyceride content as their term siblings. Previously reported differences could thus partly be due to genetic or environmental characteristics shared between siblings. The VLBW group displayed less unsaturation in subcutaneous abdominal adipose tissue, suggesting differences in its metabolic activity and energy storage.

## Introduction

Preterm birth is a major contributor to later health outcomes and risk factors. Globally 10% of babies are born preterm and 1–2% preterm at very low birth weight (VLBW, < 1500 g)^1,2^. Among other risk factors, adults born preterm at VLBW present with higher blood pressure, impaired glucose tolerance, lower bone mineral density, and they lead a more sedentary lifestyle than their term counterparts^3–6^.

VLBW adults tend to be shorter in stature^7^ than individuals born at term, while their body-mass index (BMI) is similar^8,9^. Extremely low birth weight (ELBW, < 1000 g) or VLBW adults are reported to have a lower lean body mass^10^ which, however, may be metabolically more active as indicated by higher resting energy expenditure per unit lean body mass^11,12^. The association of adverse body composition outcomes between ELBW/VLBW and term controls is more pronounced with a lower birth weight, and this difference seems to decrease with age^13–16^. Being born small for gestational age (SGA) has been linked to increased adiposity in childhood and adulthood, suggesting those born VLBW and SGA may be at particular risk^17,18^.

Abdominal adipose tissue (AT) can be roughly divided into two distinct pools: visceral (VAT) and subcutaneous abdominal adipose tissue (SAT), the two being metabolically and functionally different. An accumulation of VAT is associated with poorer metabolic and cardiovascular outcomes^19^, whereas the role of SAT accumulation regarding these outcomes is less clear, with some sources further drawing a distinction between deep (dSAT) and superficial (sSAT) subcutaneous abdominal adipose tissue^20,21^. Little is known about how prematurity affects adult adipose tissue composition, but adults born at ELBW/VLBW have been reported to display aberrant ectopic fat deposition, accumulating mostly in adipose tissue but also in muscle, epicardium and liver^22–24^. Some evidence links ELBW/VLBW to an increase in adipose tissue volume, VAT and/or SAT in childhood and early adulthood, especially in men^9,25,26^, but the reported results are heterogeneous, possibly due to methodological differences.

Excess energy may be stored as fat in other organs besides adipose tissue, often with detrimental effects. The dynamics of fat metabolism and the accumulation of fat into different depots are not yet completely understood, but a higher degree of unsaturation in SAT has been observed in obesity and is associated with an increase in intramyocellular lipid content and adverse metabolic characteristics^27^. Unsaturation, in this context, means double bonds in fatty acid carbon chains in triglycerides. An increased hepatic triglyceride content (HTGC) is associated with poor health outcomes and metabolic syndrome. Steatosis, the abnormal retention of fat in the liver, often occurs with obesity and impaired glucose metabolism, and may in time lead to steatohepatitis and eventually to cirrhosis. Evidence regarding the prevalence of non-alcoholic fatty liver disease or HTGC in VLBW adults is scant, but previous findings suggest low birth weight to be associated with an increased risk for non-alcoholic fatty liver disease in childhood^28,29^ and an increase in HTGC in early adulthood^23^.

Few studies regarding abdominal adipose tissue volume have been conducted in adults born preterm^22,23^. To our knowledge, the degree of subcutaneous fat unsaturation has never been studied with magnetic resonance spectroscopy, or any other method, in individuals born preterm. Our study design is unique in its use of term siblings as controls, as previous studies regarding the effects of VLBW or prematurity on adult health have mostly used unrelated term individuals as controls, with one register-based cohort study assessing the risk for lipid disorders in adults born preterm using co-sibling analyses^30^. The sibling setting can partially account for shared environmental and genetic confounders that other settings cannot fully address. We studied whether adipose tissue depot volumes, SAT unsaturation, or HTGC differed between adults born at VLBW and their siblings born at term.

## Material and methods

### Participants

The recruitment process has been outlined in detail^31^. In brief, we recruited 79 sex-matched sibling pairs where one was born at VLBW and the other at term. For inclusion, a maximum age difference of 10 years was allowed. We identified suitable subjects from two existing cohorts (The Helsinki Study of Very Low Birth Weight Adults and the Ester Preterm Birth Study) and from the Finnish Medical Birth Register. All VLBW subjects were born between 1978 and 1991. Exclusion criteria included pregnancy, endocrine disorders that might affect measurements, gross neurosensory or motor disorders, ongoing peroral steroid treatment, and not actually fulfilling the inclusion criteria (sibling born preterm).

**Figure 1:**
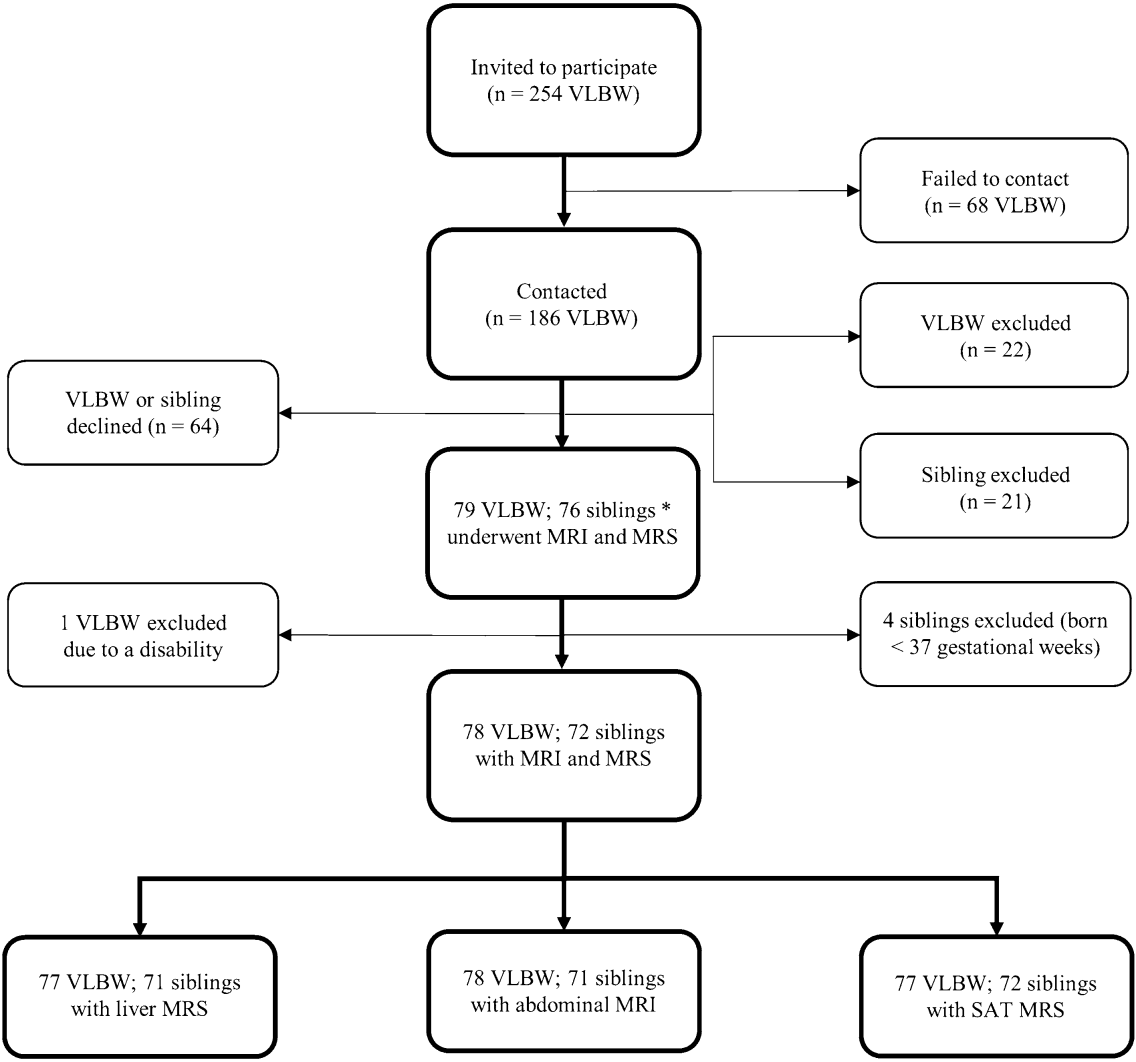
Flowchart of the data collection process. VLBW: very low birth weight, < 1500 g. MRI: magnetic resonance imaging. MRS: magnetic resonance spectroscopy. SAT: subcutaneous abdominal adipose tissue. * three siblings withdrew initial consent.

Magnetic resonance (MR) imaging was conducted between June 2014 and June 2017 as part of a comprehensive clinical assessment lasting three days. The participants underwent anthropometric measurements and completed questionnaires regarding family history, lifestyle, medications, and health during the clinical study visits. Perinatal and pregnancy information was collected from hospital and antenatal visit records. After data collection, four siblings were excluded as their birth records revealed a gestational age of less than 37 weeks. Two participants dropped out of the study without completing all study visits due to becoming pregnant after signing consent, and one participant was excluded due to a disability that was not apparent in the recruiting phase. Three term siblings withdrew before giving consent but their VLBW siblings still participated. A total of 150 suitable participants (78 VLBW and 72 term siblings) underwent abdominal MR imaging. The data collection pipeline is outlined in Fig. 1.

### Ethics

The Coordinating Ethics Committee of the Hospital District of Helsinki and Uusimaa approved the study protocol and all participants signed informed consent. The study was conducted in accordance with the Declaration of Helsinki. All images were inspected by an experienced radiologist, incidental findings were reported to the participants as stated in the study protocol, and participants were referred to further medical attention when required.

### Imaging

We used a 3.0 T MR imager (Verio, Siemens) with a body coil for abdominal imaging and a knee coil positioned distally from the knee joint to collect sufficient dimensions from the shin and calf regions. We instructed the subjects to abstain from eating and drinking for 4 h beforehand and to avoid consumption of alcohol, sauna, and strenuous exercise for two days prior to imaging. The imaging took place during weekends at any time or weekdays between 8 and 12 pm. A stack of T1-weighted MR images was acquired from the abdomen centered on the L4/L5 intervertebral disc with frequency selective fat excitation (slice thickness 10 mm, TR of 91 ms, TE of 5.2 ms and flip angle of 80°). Coronal and sagittal routine images were acquired to assist in voxel placement for magnetic resonance spectroscopy (MRS).

#### Liver MRS

**Figure 2:**
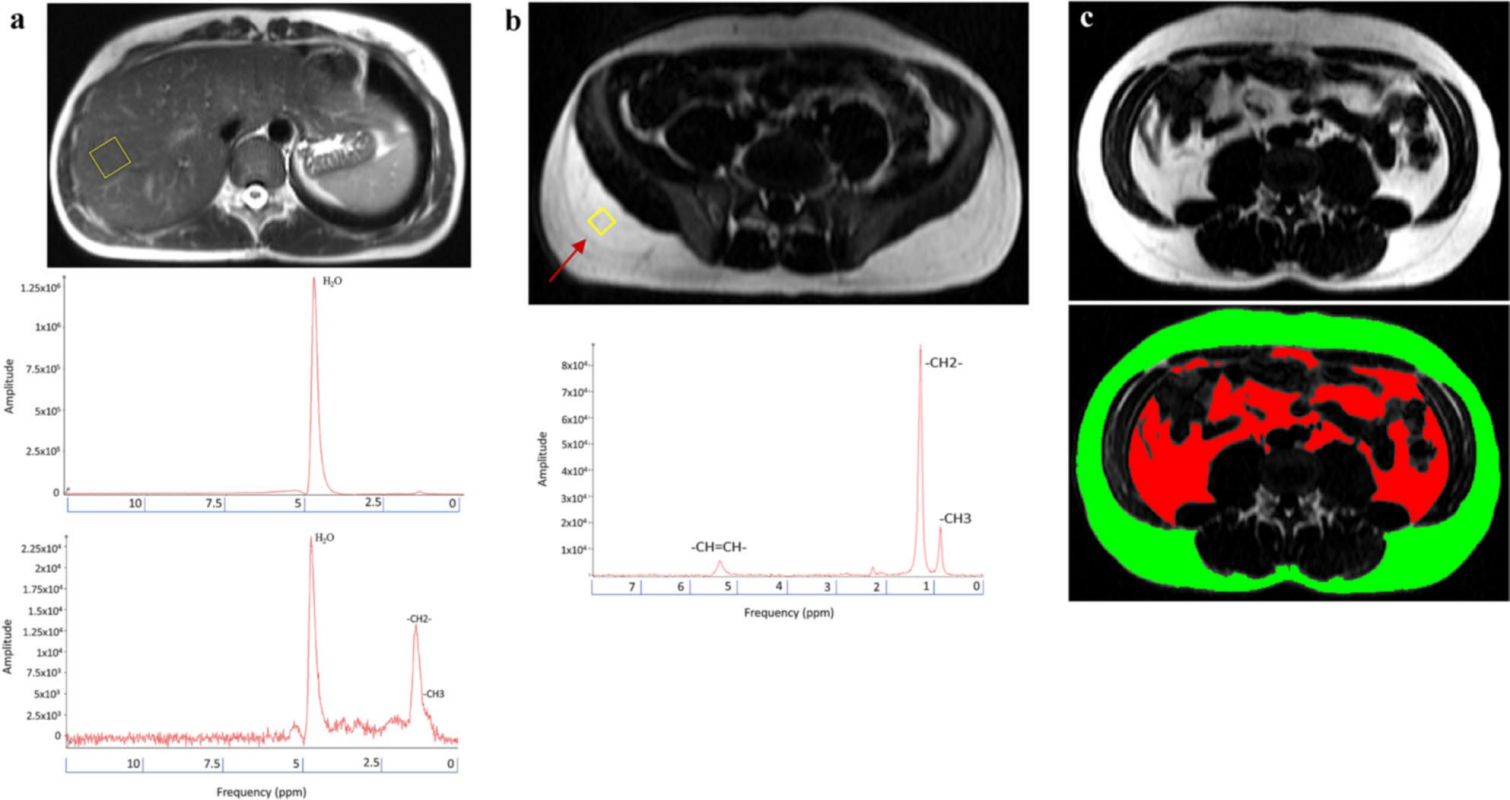
T1 weighted abdominal MR images for volumetry and magnetic resonance spectroscopy (different subjects). (**a**) A view of the positioning of the voxel (yellow box) for hepatic magnetic resonance spectroscopy avoiding large vessels and bile ducts. Spectra are displayed as with and without water suppression with a TE of 30 ms. H_2_O: water protons, –CH2: methylene protons, –CH3: methyl protons. (**b**) A view of the positioning of the voxel (yellow box) for magnetic resonance spectroscopy of subcutaneous abdominal adipose tissue depot. Care was taken to position the voxel away from Scarpa’s fascia (arrow). Spectra were collected with a TE of 200 ms. –CH = CH–: olefinic protons/double bond resonance, –CH2–: methylene protons, –CH3: methyl protons. (**c**) A Sliceomatic slice with visceral abdominal adipose tissue (red) and subcutaneous abdominal adipose tissue (green).

A 20 mm^3^ voxel cube was positioned to the deep parts of the right liver lobe within the parenchyma. Care was taken to avoid blood vessels and bile ducts as well as placing the voxel to a homogenous area while also avoiding subcutaneous and other extrahepatic contaminants. Shimming was conducted automatically by the imager software and then manually improved to achieve optimal spectral resolution. Two spectra were collected from the liver: one with water suppression and the other without. Both were triggered to the respiratory cycle and had a minimum TR of 3000 ms and a TE of 30 ms with 4 averages for the unsuppressed spectrum and 12 for the water-saturated one (Fig. 2a).

#### SAT MRS

A 12 mm^3^ voxel cube was positioned in subcutaneous adipose tissue posterolaterally at the iliac crest level ensuring that sufficient adipose tissue was present and adjusting the location accordingly. The voxel was placed below Scarpa’s fascia, which delineates the subcutaneous tissue into superficial and deep compartments. The spectra were collected with a TR of 3000 ms and a TE of 200 ms with 32 averages (Fig. 2b).

#### Bone marrow MRS

A cuboid voxel of 25 mm × 5 mm × 5 mm was positioned in the bone marrow of the proximal tibia. Care was taken to avoid cortical bone and to visually inspect any aberrations in the homogeneity of the marrow. The spectra were collected with a TR of 4000 ms and a TE of 200 ms with 32 averages.

### MRS analysis

jMRUI 6.0 beta was used for analysing MRS data: spectral quality was visually inspected for resonance integrity and resonances. Quantification was completed by jMRUI’s AMARES algorithm. All residuals were scrutinized after quantification to ensure sufficient quality of the analysis.

#### Liver

H_2_O resonances were quantified from unsuppressed spectra and the methylene and methyl resonances were primarily quantified from the water-suppressed spectra, and in cases of data quality issues from the unsuppressed spectra. A Lorentzian peak shape was used for all resonances, and soft constraints were used to correctly identify water and methylene resonances. The methyl resonance’s frequency was fixed to the methylene resonance. T2- and proton density (PD)-relaxation corrections were conducted as per in-house standards. HTGC was calculated as the ratio between the T2- and PD-corrected methylene signal and the sum of methylene and water signals.

#### SAT and bone marrow

Methylene, methyl, and olefinic/double-bond resonances were quantified using a single Lorentzian peak shape. Prior knowledge was used to facilitate identifying the correct resonances. SAT double bond content was estimated using the ratio between the double bond signal and the sum of the complete signal. Similar resonance identification and quantification methods were used for bone marrow spectra.

### AT quantification

Abdominal adipose tissue was quantified using the commercially available program SliceOmatic (Tomovision, Magog, Canada) from 16 consecutive transversal abdominal T1-weighted images centered on the intervertebral space between the fourth and fifth lumbar vertebrae. Subcutaneous and visceral adipose tissue pools were quantified using the semiautomatic ‘region growing’-tool of SliceOmatic with manual corrections using anatomical landmarks (Fig. 2c). The internal muscular wall of the abdominal cavity was used to delineate the external boundary of the VAT pool, while excluding intermuscular and paravertebral adipose tissue^20^. Accordingly, the SAT pool was labeled by being the adipose tissue external to the muscular wall of the abdominal cavity.

### Statistical analyses

All statistical analyses were conducted using IBM SPSS (version 27). Categorical variables were compared using Fisher’s exact tests or Chi-squared tests. Logarithmic conversion was used for outcome variables not following a normal distribution. Continuous variables were analyzed with paired t-tests when comparing pairs and unpaired t-tests when comparing groups. A difference of *p* < 0.05 was considered statistically significant. We used linear mixed models to assess the effect of VLBW-status on our outcomes with subjects nested within families with maximum likelihood-method. We used the following variables as fixed effects: model 1 adjusts for age and sex, model 2 further adjusts for maternal smoking, gestational hypertension, maternal BMI, and primiparity, and model 3 further adjusts for subject’s own BMI. Data on maternal smoking were available for 95% (n = 142) of all subjects, and the variables were dummy coded for two variables (1 = smoking; 0 = nonsmoking or unknown, and conversely 1 = nonsmoking; 0 = smoking or unknown) for linear mixed model analyses. Maternal BMI was available for 97% of all subjects (n = 146), and unknown data were imputed using linear regression of maternal BMI difference and maternal age difference between pregnancies. Gestational hypertension classes were defined as described previously^32^.

## Results

The VLBW group were born at a mean gestational age of 29.6 weeks and the sibling group at 39.8 weeks, with the VLBW having more variability (SD 2.5 vs. 1.3). Being born small for gestational age (birth weight < −2 SD) was more common in the VLBW group: 38.5% vs 2.8%. The groups did not differ in terms of primiparity, maternal age, maternal BMI or maternal smoking. By design, highest attained parental education was identical between groups. There was a higher incidence in gestational hypertensive disorders in the VLBW group. The characteristics of the groups are summarized in Table 1.

**Table 1:** Demographic and anthropometric characteristics of VLBW subjects and their term siblings.

	N 150 ( 53% women)				***p***
	VLBW group (n = 78)		Sibling group (n = 72)		
	Mean (range)	SD	Mean (range)	SD	
**Neonatal characteristics**					
Gestational age (wk)	29.6 (23.9–36.4)	2.5	39.8 (37.0–42.1)	1.3	< 0.001
Birth weight (g)	1150 (640–1500)	221	3390 (2100–4470)	431	< 0.001
SGA (n, %)	29 (37.2%)		2 (2.8%)		< 0.001
Had an older sibling when born (n, %)	49 (62.8%)		48 (66.7%)		0.62
**Family characteristics**					
*Highest attained parental education (%)*					
Lower secondary or lower	0%				
Higher secondary	38.6%				
Tertiary	61.4%				
Maternal age at birth (y)	29.7	4.9	30.1	5.0	0.57
Maternal BMI (kg/m^2^) (n = 146)	22.5	4.2	22.6	4.2	0.86
*Gestational hypertension (n, %)*					
Non-hypertensive	50 (64.1%)		47 (65.3%)		0.88
Gestational and chronic hypertension	4 (5.1%)		18 (25.0%)		0.001
Pre-eclampsia (PE) and superimposed PE	21 (26.9%)		1 (1.4%)		< 0.001
Proteinuria	3 (3.8%)		6 (8.3%)		0.25
Maternal smoking during pregnancy (n = 142)	11 (14.1%)		11 (15.5%)		0.72
**Adult participant characteristics**					
Age (y)	29.4	2.6	29.1	4.9	0.72
Height women (cm) Height men (cm)	162.3 174.0	7.1 7.8	165.7 180.0	5.5 6.9	0.02 0.001
Weight women (kg) Weight men (kg)	63.4 75.4	15.4 12.8	65.3 83.6	15.1 14.6	0.57 0.02
BMI (kg/m^2^) women BMI (kg/m^2^) men	24.0 24.9	5.4 3.9	23.7 25.7	5.0 3.9	0.81 0.37

VLBW: very low birth weight (< 1500 g).

SGA: small for gestational age (< − 2 SD).

BMI: body mass index.

Liver spectra were available from 77 VLBW subjects and 71 term controls, with one sibling pair not completing liver imaging due to a congenital disorder affecting liver morphology. SAT spectra were available from 77 VLBW subjects and 72 term controls with one MRS collection failing due to a malfunction in the imager. Bone marrow spectra were available for 65 VLBW subjects and 60 siblings. The number for subjects with bone marrow MRS available was slightly lower than the other outcomes due to its incorporation into the study protocol after a pilot phase. Abdominal MR images of sufficient quality were available from 78 VLBW subjects and 71 siblings. One set of images was of an inferior resolution due to technical issues with the imager, and was thus excluded from the analyses.

When comparing the volumetric outcome variables in unadjusted paired t-tests (70 whole pairs), the abdominal adipose tissue results were similar between VLBW subjects and their siblings: total abdominal adipose tissue (3719 ml vs 3801 ml, *p* = 0.54), subcutaneous abdominal adipose tissue (3002 ml vs 3100 ml, *p* = 0.48) and visceral intra-abdominal adipose tissue (656 ml vs 644 ml, *p* = 0.79). The spectroscopic results were also similar. Unadjusted T2- and PD-corrected mean hepatic triglyceride content was 1.37% in the VLBW group and 1.33% in the sibling group (*p* = 0.94). Neither did the double bond ratio of subcutaneous abdominal adipose tissue reach statistical significance (9.75% vs 10.09%, *p* = 0.20), and the double bond ratio of proximal tibial bone marrow was also similar between groups (12.30% vs 12.29%, *p* = 0.91). The unadjusted results are summarized in Table 2.

**Table 2:** Abdominal adipose tissue volumes, liver fat, and fat unsaturation in the VLBW and term-born sibling control groups.

	VLBW subjects	Term siblings	*p* value
	Mean (GSD)	Mean (GSD)	
**Adipose tissue volume (ml)**			
Total adipose tissue	3719 (1.72)	3801 (1.63)	0.54
Subcutaneous adipose tissue	3002 (1.73)	3100 (1.61)	0.48
Visceral adipose tissue	656 (1.95)	644 (1.95)	0.79
**Magnetic resonance spectroscopy (%)**			
Hepatic triglyceride content	1.37 (3.00)	1.33 (2.87)	0.94
Subcutaneous adipose tissue unsaturation	9.75 (1.02)	10.09 (1.02)	0.20
Bone marrow unsaturation	12.30 (1.01)	12.29 (1.01)	0.91

VLBW: very low birth weight (< 1500 g).

Numbers represent geometric means and standard deviations. *p* values for differences between VLBW subjects and controls calculated with paired samples t-tests. Abdominal adipose tissue pool volumes are presented in millilitres, hepatic triglyceride content is presented as the ratio between the sum of T2- and PD-corrected methylene and water resonances in the liver, and subcutaneous and bone marrow unsaturation are presented as the ratio between the double bond and methylene resonances in subcutaneous adipose tissue and proximal tibial bone marrow, respectively.

**Figure 3:**
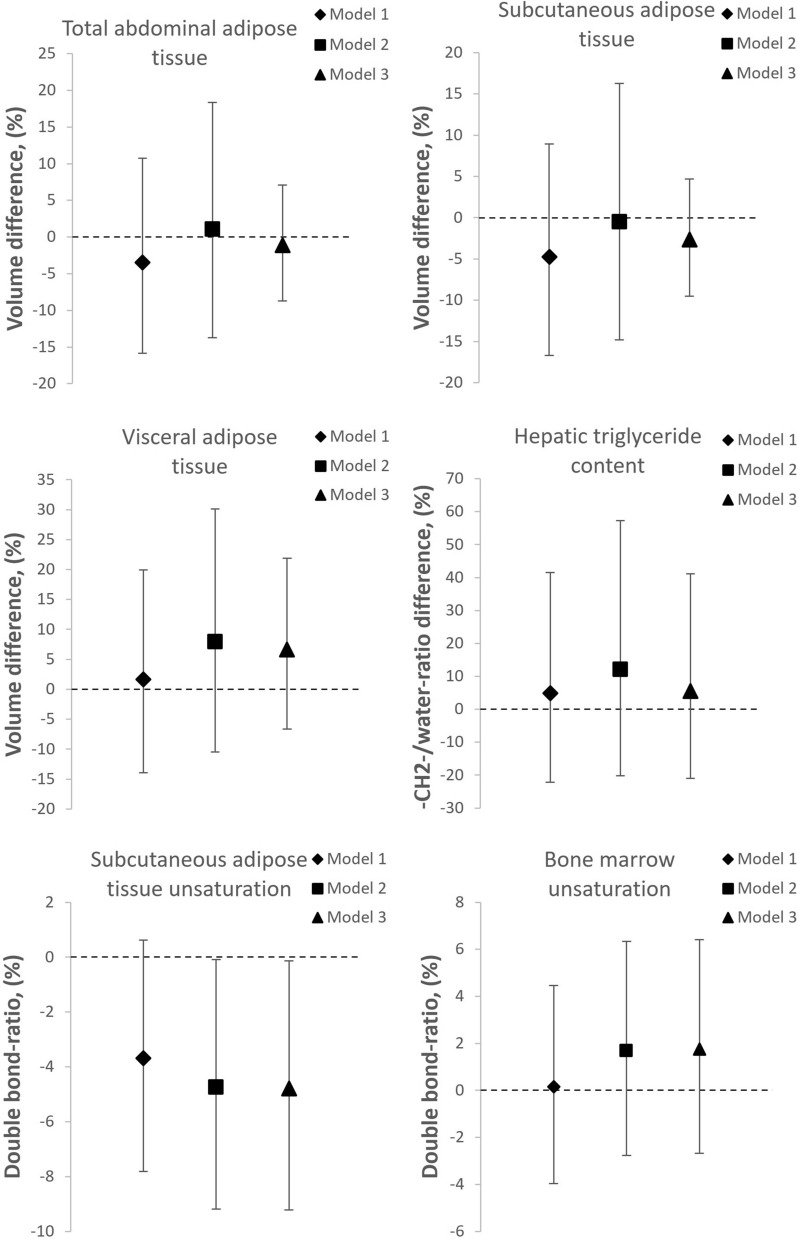
Mean differences (95% CI error bars) of log-transformed volumes of visceral, subcutaneous and total abdominal adipose tissue, hepatic triglyceride content, and subcutaneous adipose tissue and bone marrow unsaturation in adults born at VLBW compared to their term born siblings (zero line). Model 1 adjusted for sex and age at examination. Model 2 further adjusted for primiparity, maternal age, maternal BMI, maternal smoking, gestational hypertension and pre-eclampsia. Model 3 further adjusted for current BMI.

To account for confounders and within-family- effects we used linear mixed models after logarithmic conversion of the non-normally distributed outcome variables. All results in the following paragaraph are from model 2, which adjusts for sex, age, maternal BMI, maternal smoking during pregnancy, gestational hypertension, and primiparity, unless stated otherwise. The results are summarized in Fig. 3 and presented in detail in Table 3.

**Table 3:** Fixed effect estimates indicating mean %-differences between 78 VLBW adults and 72 sibling controls born at term, adjusted for covariates.

	Effect estimate (%)	95% CI lower limit (%)	95% CI upper limit (%)
Abdominal adipose tissue**			
Model 1	− 3.44	− 15.83	10.76
Model 2	1.05	− 13.72	18.36
Model 3	− 1.11	− 8.69	7.10
Subcutaneous adipose tissue**			
Model 1	− 4.72	− 16.67	8.96
Model 2	− 0.48	− 14.80	16.26
Model 3	− 2.67	− 9.50	4.68
Visceral adipose tissue**			
Model 1	1.63	− 13.90	19.96
Model 2	7.96	− 10.44	30.14
Model 3	6.68	− 6.62	21.87
**Hepatic triglyceride content (n = 77 + 71)**			
Model 1	4.94	− 22.21	41.58
Model 2	12.05	− 20.16	57.26
Model 3	5.61	− 20.95	41.08
**Subcutaneous fat double bond ratio (n = 77 + 72)**			
Model 1	− 3.69	− 7.82	0.62
Model 2	− 4.74*	− 9.19	− 0.08
Model 3	− 4.79*	− 9.22	− 0.14
**Bone marrow double bond ratio (n = 65 + 60)**			
Model 1	0.16	− 2.96	3.38
Model 2	1.68	− 1.86	5.35
Model 3	1.76	− 1.78	5.42

VLBW: very low birth weight (< 1500 g).

Outcomes were log-transformed for analyses and are presented as %-differences using linear mixed models.

Model 1 is adjusted for sex and age.

Model 2 further adjusts for primiparity, maternal age, maternal BMI, maternal smoking, and gestational hypertension and pre-eclampsia.

Model 3 further adjusts for subject’s BMI.

*Denotes a statistical significance of *p* < 0.05.

**Data available from 78 VLBW subjects and 71 siblings.

The VLBW group did not differ from the term sibling group regarding adipose tissue volumes: AT (1.05%, 95% CI − 13.72%, 18.36%), SAT (− 0.48%, 95% CI − 14.80%, 16.26%) and VAT (7.96%, 95% CI − 10.44%, 30.14%). In the outcomes measured by MRS, we saw no differences in hepatic triglyceride content (12.05%, 95% CI − 20.16%, 57.26%) or double bond ratio of the bone marrow in proximal tibia (1.68%, 95% CI − 1.86%, 5.35%). We did, however, observe a lower ratio of double bonds indicating a lesser degree of unsaturation in subcutaneous abdominal adipose tissue (− 4.74%, 95% CI − 9.19%, − 0.08%). Further adjustment for the participant’s BMI (model 3, Table 3) or SGA-status did not impact the results.

## Discussion

In this unique cohort of adults born preterm at VLBW and their term siblings, we found that VLBW adults did not differ from their siblings regarding volumes of visceral, subcutaneous or total abdominal fat, hepatic triglyceride content or bone marrow unsaturation. However, preterm VLBW adults had a lower degree of unsaturation in abdominal subcutaneous adipose tissue than their siblings, after adjusting for age, sex, and maternal and perinatal factors, or after further adjusting for the subject’s own BMI.

Previous studies have shown that children and adults born at VLBW display increased cardiovascular risk factors^5,7^ as well as lower lean body mass^10^. VLBW has also been linked to an increased amount of adipose tissue in some but not all studies. The findings are heterogeneous, possibly due to small sample sizes and methodological differences in literature. In a meta-analysis of 602 preterm adults (of whom 301 were very preterm/VLBW/ELBW) and 656 term controls, fat mass was 1.22%-1.46% higher in adults born preterm^3^. Studies using DXA or ultrasonography-based fat layer thickness estimation^26,33^ have reported a lower lean body mass^4,10,14^, and some^4,14^ but not all^10^ of the studies have reported a higher body fat percentage^4,14^, with some evidence suggesting a more pronounced association with a more extreme phenotype, as reported by Alves et al.^26^ comparing ELBW to VLBW children. There is also longitudinal evidence suggesting an age-dependent association of increasing adiposity with age when comparing preterm children, adolescents and adults by air-plethysmography^9^.

Few studies have assessed the distribution of abdominal adipose tissue using MRI in VLBW or ELBW adults, and even among these, methodology varies substantially making direct comparison challenging. Thomas et al.^22^ compared 23 preterm subjects (< 33 gestational weeks) with 25 term controls as young adults, and observed a larger amount of SAT and VAT in the preterm group (0.70 L; 95% CI 0.13, 1.27 and 0.51 L; 95% CI 0.1, 0.9 respectively), when measuring the whole abdominal cavity and adjusting for BMI. They also noted a sex-interaction, where the difference in VAT volume was only observable in men (preterm 2.1 (SD 1.1) vs. term 1.2 (SD 0.6)), but not in women (preterm 1.1 (SD 0.6), term 1.1 (SD 0.3); sex-group interaction *p* = 0.056). Thomas et al. also observed a larger ratio of intrahepatocellular lipids (3.07; 95% CI 1.78, 5.28) in preterm subjects using MRS, whereas we observed no difference in HTGC (12.05%, 95% CI − 20.16%, 57.26%). Of note, they reported only analyses adjusted for adult BMI. Crane et al.^23^ compared 29 ELBW subjects to 13 normal birth weight adults at a mean age of 34. Instead of adipose tissue volumes, they assessed subcutaneous and visceral fat areas by MRI from a single slice 5 cm cranially from L4/L5. Crane et al. reported ELBW individuals having more subcutaneous but not visceral fat than normal birth weight controls. They also report a higher hepatic fraction measured by LAVA-FLEX fat suppression imaging technique in the ELBW group. In a multivariate analysis, however, the group differences in fat areas or hepatic fat fraction were driven by sex and BMI—not by birth weight group.

Contrary to earlier studies that used unrelated controls, our study of same-sex siblings discovered no differences in AT, SAT or VAT volumes in paired t-tests or after adjusting for confounders. Neither did any difference appear after further adjustment for current BMI that Thomas et al.^22^ adjusted for in their analysis. This suggests that genetic or environmental confounders shared within a family could partly explain the observed differences in previous studies. The differences in adiposity might also decrease or change with age^9,14^, and in our population with a mean age of 29, the previously suggested differences of childhood^16^ or early adulthood^13,22^ might no longer have been present. This reasoning may account for our observed similarity between groups in hepatic triglyceride content.

This study is, to our knowledge, the first one to assess the degree of unsaturation in subcutaneous abdominal adipose tissue in VLBW adults. Measuring unsaturation from different adipose tissue pools has been validated by gas chromatography^34^, and is commonly used in metabolic imaging in various organs and tissues. Greater unsaturation in subcutaneous tissue has been associated with obesity and insulin resistance in twin studies^27^ From this perspective, our results of a lower unsaturation degree in VLBW adults seem counterintuitive. We also measured bone marrow unsaturation to assess whether any differences in unsaturation would be global, but we saw no differences between groups. Bone marrow unsaturation has been reported to be an independent process from obesity^35^. It could be speculated that a higher resting energy expenditure would result in differences in adipose tissue metabolism and thus unsaturation, possibly due to less surplus energy being available for storage as unsaturated fatty acids^11^. Further studies, however, are needed to investigate how adipose tissue unsaturation, metabolic outcomes, fat composition, and energy expenditure link to each other. Together, our bone marrow and SAT MRS findings suggest that the unsaturation may be specific to adipose tissue or fat metabolism in general, but this remains to be confirmed, and potential adipose tissue biopsy verification could provide clarity.

Our sibling study protocol provided both strengths and limitations. It allows partial circumvention of unmeasured shared familial confounders that traditional case–control-studies cannot fully address. On the other hand, our study design limits participation to VLBW adults who have a same-sex sibling willing to participate in an assessment that included three separate study visits. While the participants could decide whether to participate during the same or separate visits than their siblings, virtually all chose to attend the visits together. It is possible that our study population has an overexpression of sibpairs who feel close to each other and who could more likely share other characteristics including lifestyle. Another limitation is the maximum age difference of 10 years between siblings, during of which environmental conditions may have changed. It would also be of interest to see how dietary and nutritional factors affect abdominal adipose tissue in future studies. Our sample size is large both compared to previous studies of fat compartments/ectopic fat, and MRI-based studies assessing adipose tissue volumes in VLBW adults, which lends robustness to both our negative and positive results. Our subjects are also older than in many cohorts examining late health outcomes of prematurity. To our knowledge, no previous study has ever assessed adipose tissue unsaturation in VLBW adults, making our study inaugural. A possible limitation is our measurement of unsaturation from dSAT, as some evidence suggests that the unsaturation of sSAT and dSAT may differ.

## Conclusion

Adults born preterm at VLBW did not differ from their term-born same-sex siblings regarding abdominal adipose tissue volumes, hepatic triglyceride content or bone marrow unsaturation. This suggests that previously reported differences could partly be due to genetic or environmental characteristics shared within a family. VLBW adults did, however, display a lower degree of unsaturation in their abdominal subcutaneous adipose tissue, which might be related to differences in adipose tissue metabolic activity.

## Data Availability

The datasets generated during and/or analysed during the current study are not publicly available due to them containing individual level data, even though the data are anonymized. The datasets are available from the corresponding author on reasonable request.

## Abbreviations

VLBW: Very low birth weight, < 1500 g
ELBW: Extremely low birth weight, < 1000 g
LBW: Low birth weight, < 2500 g
SGA: Small for gestational age (< − 2 SD)
AT: Abdominal adipose tissue
VAT: Abdominal visceral adipose tissue
SAT: Abdominal subcutaneous adipose tissue
dSAT: Deep subcutaneous adipose tissue
sSAT: Superficial subcutaneous adipose tissue
HTGC: Hepatic triglyceride content
MR: Magnetic resonance
MRS: Magnetic resonance spectroscopy
BMI: Body mass index
